# Can Playing the Computer Game “Tetris” Reduce the Build-Up of Flashbacks for Trauma? A Proposal from Cognitive Science

**DOI:** 10.1371/journal.pone.0004153

**Published:** 2009-01-07

**Authors:** Emily A. Holmes, Ella L. James, Thomas Coode-Bate, Catherine Deeprose

**Affiliations:** Department of Psychiatry, University of Oxford, Oxford, United Kingdom; King's College London, United Kingdom

## Abstract

**Background:**

Flashbacks are the hallmark symptom of Posttraumatic Stress Disorder (PTSD). Although we have successful treatments for full-blown PTSD, early interventions are lacking. We propose the utility of developing a ‘cognitive vaccine’ to prevent PTSD flashback development following exposure to trauma. Our theory is based on two key findings: 1) Cognitive science suggests that the brain has selective resources with limited capacity; 2) The neurobiology of memory suggests a 6-hr window to disrupt memory consolidation. The rationale for a ‘cognitive vaccine’ approach is as follows: Trauma flashbacks are sensory-perceptual, visuospatial mental images. Visuospatial cognitive tasks selectively compete for resources required to generate mental images. Thus, a visuospatial computer game (e.g. “Tetris”) will interfere with flashbacks. Visuospatial tasks post-trauma, performed within the time window for memory consolidation, will reduce subsequent flashbacks. We predicted that playing “Tetris” half an hour after viewing trauma would reduce flashback frequency over 1-week.

**Methodology/Principal Findings:**

The Trauma Film paradigm was used as a well-established experimental analog for Post-traumatic Stress. All participants viewed a traumatic film consisting of scenes of real injury and death followed by a 30-min structured break. Participants were then randomly allocated to either a no-task or visuospatial (“Tetris”) condition which they undertook for 10-min. Flashbacks were monitored for 1-week. Results indicated that compared to the no-task condition, the “Tetris” condition produced a significant reduction in flashback frequency over 1-week. Convergent results were found on a clinical measure of PTSD symptomatology at 1-week. Recognition memory between groups did not differ significantly.

**Conclusions/Significance:**

Playing “Tetris” after viewing traumatic material reduces unwanted, involuntary memory flashbacks to that traumatic film, leaving deliberate memory recall of the event intact. Pathological aspects of human memory in the aftermath of trauma may be malleable using non-invasive, cognitive interventions. This has implications for a novel avenue of preventative treatment development, much-needed as a crisis intervention for the aftermath of traumatic events.

## Introduction

We suggest that basic principles from cognitive science may be used to help develop an intervention for trauma flashbacks, and propose a ‘cognitive vaccine’ approach. That is, that the delivery of specific cognitive tasks may help ‘inoculate’ against the escalation of flashbacks after a traumatic event. Post Traumatic Stress Disorder (PTSD) is a psychiatric disorder that can result from experiencing or viewing a traumatic event involving death, serious injury, or threat to self or others [1], [2]. A precursor [3] and indeed the hallmark symptom of PTSD [1] is vivid flashbacks to the trauma, that is, distressing, re-experiencing of the trauma in the form of intrusive, image-based, sensory-perceptual memories. For example, following a motor vehicle accident, a person may later experience intrusive flashbacks where in their mind's eye they suddenly see a vision of a looming car accompanied by the sound of crashing metal.

Although we have successful treatments for full-blown PTSD, crisis interventions to reduce the build up of symptoms in the early aftermath of trauma are lacking. Current interest in the manipulation of memories post-trauma is particularly focused on pharmacological means, for example, propranolol administration [4]. In addition to the potential for side-effects with pharmacological approaches, there are potential ethical concerns if voluntary memories for human experience are suppressed [5]. For example, removing flashbacks at the expense of being able to deliberately remember what happened during a trauma could compromise a trauma victim's ability to testify in court. We have also raised clinical concern over treatment innovations stemming from exciting theoretical developments [6], [7] but which advocate psychological approaches which promote the suppression of memory for traumatic experiences as way of dealing with negative sequelae, since suppression is clinically contra-indicated [8], [9].

The psychological intervention with the strongest evidence-base for full-blown PTSD is trauma-focussed Cognitive Behaviour Therapy–a treatment which is only indicated when delivered weeks or months after the trauma [10]. However, what can be given to trauma victims suffering flashbacks in the first few weeks? Unfortunately, using talking therapy as a crisis intervention in the immediate aftermath of trauma has caused international clinical concern [11]: interventions such as critical incidence stress debriefing can worsen rather than ameliorate later trauma symptoms [12]. Given the scale of traumatic events globally–war, terrorism, natural disasters, interpersonal violence–there is a huge unmet need for widely-available and easily accessible interventions. We need to develop fresh theory-driven interventions to reduce the build up of flashbacks in the early post-trauma period.

Can cognitive science suggest a way to reduce the build-up of flashbacks to trauma? We suggest that certain cognitive tasks, informed by the neuropsychological domain of working memory, may indeed be used tap into processes underlying flashback memory consolidation. This is based on two key findings: first, cognitive science has shown the brain has selective resources with limited capacity [13]; second, the neurobiology of memory suggests there is a 6-hr window to disrupt memory consolidation [14], [15]. In particular, we suggest that visuospatial tasks will be useful in this regard according to the following rationale: (1) trauma flashbacks are sensory-perceptual images with visuospatial components [2], [16], [17]. (2) visuospatial cognitive tasks compete for resources with visuospatial images [18]–[21]. (3) the neurobiology of memory consolidation suggests a 6-hr time frame post-event within which memories are malleable [14], [15]. Thus (4) we predict visuospatial cognitive tasks given within 6-hr post-trauma will reduce flashbacks.

Following a long tradition in experimental psychopathology, we use the trauma film paradigm [22] in our laboratory as an experimental analog of viewing real trauma and the subsequent flashbacks [23], [24] suffered in PTSD. We have previously demonstrated that the frequency of flashbacks for analog traumatic content can be manipulated experimentally by completing standardized cognitive tasks *during* trauma film viewing (i.e. peri-traumatically): In healthy participants, completing a complex visuospatial working memory task [19], such as finger pattern tapping, during exposure to a trauma film subsequently reduced flashbacks of that film over 1-week compared to no task, or non-visuospatial tapping [24], [25]. Interestingly, and in line with cognitive theories of PTSD [2], verbal cognitive tasks during the film, such as counting backwards in threes, actually *increased* the number of flashbacks, confirming that the effects were not simply due to distraction for general working memory resources but to the specific nature of the task [24].

In the current study it was critical to identify a visuospatial working memory task that would be widely-available in the real-world. Clearly a standard neuropsychological test battery visuospatial task such as the WAIS block design [26] would be impractical to deliver en mass. The popular computer game “Tetris” has been demonstrated to be a visuospatial task [27]–[30], drawing on mental rotation and the type of processing we recruit when forming mental images. Playing “Tetris” can even result in participants experiencing subsequent visual images of the game itself at a later time [30], implicating its involvement with intrusive, image-based memory. The capacity of visual memory is both limited and vulnerable to proactive interference, i.e. interruption of memory for presented stimuli by the presentation of similar, but different stimuli after a time delay [31]. Thus “Tetris” provides a promising candidate. Interestingly, watching violent films and playing prolonged computer games are typically associated with negative impact on psychological well being and behavior in both children and adults [32]. This has led to public concern over their ready availability. However, clearly not all computer games are bad for you [27].

We now report the impact of a cognitive visuospatial task, a computerized mental rotation game, on the modulation of analog flashbacks to trauma. Given our earlier experimental demonstration that engaging in visuospatial tasks peri-traumatically (i.e. during trauma film viewing) reduces subsequent intrusive imagery (analog flashbacks), we tested here whether the window for intervention could be extended into the post-trauma period (i.e. after trauma film viewing). This is a critical question since in real-world trauma successful manipulation of flashbacks would need to be conducted post-event rather than peri-traumatically. Further, investigating a time-frame for intervention in the near aftermath of trauma, yet within a window of memory malleability has clear clinical implications. Recent statistics indicate that the average waiting time in an emergency department in the United States is 30-mins [33]. To test the real-world application of our paradigm, we explored the manipulation of flashbacks in the laboratory 30-min after watching a trauma film.

Our suggestion to manipulate flashbacks in the aftermath of a traumatic experience is supported by various experimental demonstrations that newly formed memories are initially labile for a short time and thus subject to interference. In rats, the consolidation of fear memories can be inhibited in a dose dependent fashion with administration of either anisomycin or Rp-cAMPS in the immediate post-training phase [34]. Furthermore, even established memories may be subject to manipulation upon reactivation–the triggering of previously consolidated memories may return them to a labile state and in need of *reconsolidation* if they are to persist [14], [35], [36]. In humans, the consolidation or stabilization of motor memory (in this case, a finger pattern tapping task) has been demonstrated to occur in the first 6-hours following initial learning: learning a new variation on the motor task after reactivation of the previously learned motor task can block the reconsolidation of memory for the original task [15]. Although there are doubts as to whether such reconsolidation may occur for all types of memory [37], of particular interest to PTSD research is that flashbacks for trauma may be pharmacologically modulated [4], [38], though this has not been without controversy [5]. Given the potential advantages of a non-invasive procedure, we were interested in testing whether a cognitive intervention-a visuospatial task-could modulate later flashbacks to traumatic material.

We predicted that playing a visuospatial computer game requiring mental rotation of shapes (“Tetris” [39]), 30-min after viewing graphic and traumatic film footage, would help reduce later involuntary flashbacks of the traumatic material, but leave voluntary memory retrieval intact. Similarly we predicted that playing “Tetris” would be associated with reduced clinical symptomatology at one week.

## Results

**Figure 1:**
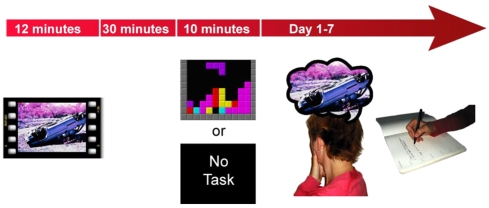
Study design overview. Participants completed a well-established experimental analog for PTSD, the trauma film paradigm. All participants viewed a traumatic film consisting of scenes of real injury and death and had a 30-min structured break. Participants were randomly allocated to either a no-task or visuospatial condition which they undertook for 10-min. Flashbacks (involuntary memories) were monitored for 1-week using a daily diary. Then participants returned to the laboratory for an assessment of clinical symptomatology relating to the flashbacks as well as a test of voluntary memory (recognition memory).

Forty participants watched a 12-min film of traumatic scenes of injury and death (n = 20 per group). Film viewing was followed by a 30-min interval before simple random assignment to one of two experimental conditions (Figure 1). There were no baseline differences between the two groups in terms of age, depressive symptoms or trait anxiety (Table 1) or gender. Mood was equivalent between the groups prior to watching the film, and as predicted, both groups experienced comparable mood deterioration following the film (Table 2).

**Table 1:** Means and statistics for age and baseline assessments indicating experimental groups were equivalent at baseline.

Measure	Visuospatial (n = 20)		No-task (n = 20)		t-test
	mean	sem	mean	sem	
Age	22.4	1.4	24.4	1.1	t_(38)_ = 1.15 (NS)
Beck Depression Inventory	6.2	1.2	5.1	1.6	t_(38)_ = 0.56 (NS)
Trait Anxiety (STAI-T)	39.3	2.2	36.5	1.7	t_(38)_ = 1.00 (NS)

**Table 2:** Means and statistics for negative mood assessment (pre vs post-trauma film) indicating equivalent deterioration in mood across conditions prior to the administration of the experimental task.

	Visuospatial (n = 20)		No-task (n = 20)		ANOVA		
	mean	sem	mean	sem	Time	Group	Group*Time
Pre-Trauma Film Mood	5.1	1.0	4.3	0.8	*F*_(1, 38)_ = 26.81 §	*F*_(1, 38)_ = 0.06 (NS)	*F*_(1, 38)_ = 1.76 (NS)
Post-Trauma Film Mood	8.4	1.2	9.9	1.3			

§ p<0.01.

Following the 30-min interval period in which participants completed standardized filler tasks, a brief reminder task for the trauma film was administered to both groups. Participants then either completed the visuospatial condition or sat quietly (no-task control condition) for 10-min. During these 10-min, all participants recorded the frequency of trauma film flashbacks they experienced. Significantly fewer flashbacks were experienced in this initial period (i.e. while playing the game or not) during the visuospatial condition than the no-task condition (Table 3).

**Table 3:** Frequency of initial flashbacks during the 10-min experimental task indicating that there were significantly fewer involuntary flashback memories during the visuospatial compared to the no-task control condition.

Group	mean	sem	t-test
Visuospatial (n = 20)	4.6	1.1	t_(38)_ = 2.50 †
No-task (n = 20)	12.8	3.1	

† **= **p<0.05.

**Figure 2:**
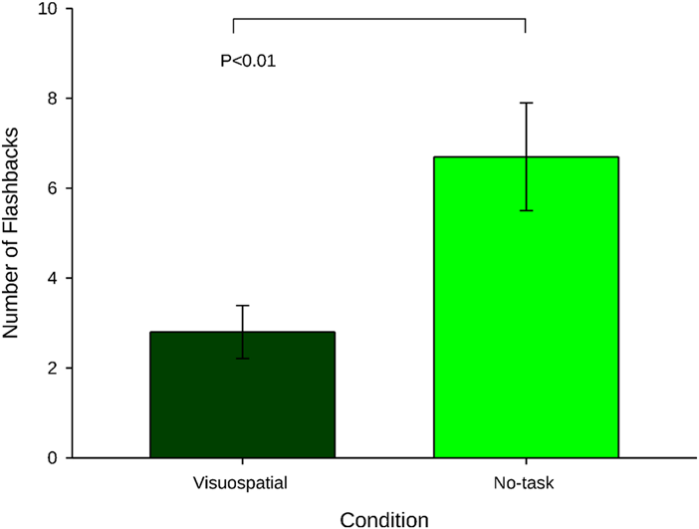
Flashback frequency over 1- week. As predicted, there was a significant reduction in the number of flashbacks over 1-week in the visuospatial condition compared to no-task condition, t_(38)_ = 2.87 (mean+/−sem).

**Figure 3:**
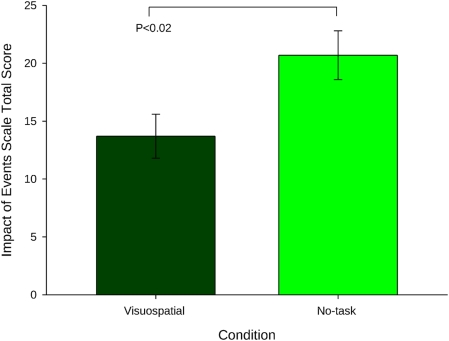
Clinical symptomatology at 1-week. Impact of Event Scale scores at 1-week indicated significantly lower impairment in the visuospatial condition compared to no-task condition, t_(38)_ = 2.47 (mean+/−sem).

After leaving the laboratory, participants then kept a daily diary in which they recorded their flashbacks to the trauma film over a period of 1-week. Crucially, we found that participants in the visuospatial condition experienced significantly fewer flashbacks over the week (Figure 2) than those in the control condition. Furthermore, at 1-week, participants returned to the laboratory–participants in the game condition had significantly lower scores on the measure of clinical symptomatology of trauma–the Impact of Events Scale [40] (Figure 3).

On a recognition memory test for the trauma film given after 1-week, performance was comparable in the visuospatial and no-task conditions (Table 4), indicating that voluntary memory for the film appeared intact. That is, completing the visuospatial condition had served to reduce only the trauma flashbacks typically associated with psychological distress and dysfunction, but not the actual memories for the events which could still be deliberately recalled.

**Table 4:** Recognition memory for trauma film after 1-week indicating equivalent voluntary memory on the recognition task across conditions.

Group	mean	sem	t-test
Visuospatial (n = 20)	20.1	0.8	t_(38)_ = 0.10, (NS)
No-task (n = 20)	20.2	0.7	

## Discussion

Our data demonstrate that recently formed sensory-perceptual memories are vulnerable to manipulation 30-min following watching a traumatic film. Why more precisely might visuospatial computer games be effective in reducing at least analog flashbacks following trauma? In accordance with the longstanding psychological model of human memory–the working memory model [13], [18]–[20], [41], [42], we propose that strategic, selective interference with the consolidation of recently triggered visual memories occurs via the demand on the player's limited visuospatial working memory resources. The major clinical theories of PTSD [2], [43], [44], [45] converge to suggest that there are two forms of processing that occur simultaneously for any given traumatic event: (1) the sensory-perceptual processing of the trauma e.g. the sights and sounds experienced during a car crash; (2) verbal or conceptual processing e.g. making sense or a coherent narrative about what is occurring. It is information from sensory-perceptual processing that provides the foundation for flashback images. Clinical models of PTSD propose that the relative balance of sensory-perceptual versus verbal/conceptual processing of a traumatic event determines whether flashbacks are formed, whereby a skewed balance towards sensory-perceptual aspects of the trauma is pathological.

When viewing traumatic film stimuli, the type of sensory-perceptual focus engaged in is predominantly visual (rather than say olfactory or auditory). To interfere with this type of visual processing we need to target what are known cognitively as ‘visuospatial’ resources [19]. That is, visuospatial tasks that use the same type of processing as do visual flashbacks will interrupt memory consolidation of those flashbacks by competition for the same limited cognitive resources [24]. Thus, by selectively interfering with visual sensory-perceptual processing of the traumatic film via visuospatially demanding cognitive tasks, subsequent analog flashbacks are reduced. Note, this is not the same as simple distraction, since other types of tasks such as verbal tasks during traumatic films are predicted (and have been shown) to lead to increased flashbacks [23], [24]. Our data is the first indication that the manipulation of visuospatial processing in the consolidation phase of recently activated trauma memories can serve to modulate future intrusive, involuntary flashbacks (despite leaving voluntary memory intact). “Tetris” participants experience fewer intrusions even while playing the game, supporting the competition for resources rationale. Significantly, we demonstrate that the visuospatial task conducted 30-min post-exposure to traumatic stimuli is effective in reducing flashbacks of that trauma as well as associated clinical symptomatology over 1-week.

Interestingly, the clinical literature offers potentially converging support for these findings. Eye Movement Densensitization and Reprocessing (EMDR) is an empirically-supported treatment for established PTSD [10]. During this therapy, the patient undergoes a series of eye movements whilst holding an intrusive traumatic memory in mind, leading to a reduction in the emotionality and vividness of the unpleasant mental image. One of several possible accounts of how EMDR might work is that the eye movements draw on visuospatial processing [18], [46], [47] and thus provide a dual task competing specifically for resources with the trauma imagery, reducing its impact. Our proposal similarly draws on a working memory rationale for how “Tetris” may affect flashback formation in a modality specific manner [48], though emphasizes all the senses involved in imagery (not just the visual modality). However, a critical difference between the current experiment and EMDR is that EMDR is used for treating existing flashbacks in PTSD (at least one month post-trauma), but is not intended to be used during the memory consolidation phase targeted in the current study. Our interest in the immediate aftermath of trauma is to understand *preventative* (rather than just curative) measures to the development of PTSD flashbacks.

Speculatively, the effects of “Tetris” may not be limited to the immediate post-trauma period during which it is played but may even continue to compete for visuospatial resources later. For example, it has already been demonstrated [30] that images of “Tetris” can intrude during sleep-a period during which memory consolidation is known to occur. Future research is required to examine the precise mechanisms of action by which “Tetris” reduces flashbacks to trauma. We predict that a verbal task would not have comparable effects and may even worsen flashbacks. Thus future studies should compare both a visuospatial task (e.g. “Tetris”) with a verbal task (e.g. a verbal computer game) against a no-task control group.

Our alternative and novel approach of using cognitive visuospatial tasks, rather than pharmacological means to reduce flashbacks following trauma aims to deal directly with the consolidation and potentially, reconsolidation, of such intrusive imagery in an ethical, safe and economical way. We suggest this approach could be harnessed as a ‘*cognitive vaccine*’ to inoculate against escalation of flashbacks contributing to full blown PTSD. Further research is required but potential clinical applications of our paradigm include use by emergency services in the early post-trauma period, e.g. to victims of rape or delivering such tasks to populations subject to regular trauma exposure e.g. firefighters or those involved in armed combat. To better map the horizons of human memory, we should further delineate the clinical possibilities offered by cognitive theory to reduce pathological aspects of memory, such as flashbacks.

## Materials and Methods

Forty participants (aged 18–47 years; mean age = 23 years; 22 males) completed baseline assessments of mood, trait anxiety and depression and then viewed traumatic film footage (Figure 1). The 12-min film contained 11 clips of traumatic content including graphic real scenes of human surgery, fatal road traffic accidents and drowning. Following the film, mood assessments were repeated and standardized filler tasks completed for 30-min. A brief film reminder task was then administered in which one neutral but recognizable image from each film clip was presented to all participants.

According to randomization to condition (simple, computer-generated random allocation), participants either completed the visuospatial condition or were in a no-task control condition for 10-min. Participants in the visuospatial condition played the game (“Tetris”) on a computer and used the cursor keys to move and rotate falling blocks to complete the largest number of complete rows across the screen. In the no-task control condition, participants were asked to sit quietly for 10-min. During both conditions participants recorded initial flashbacks of the trauma over the 10-min.

Participants then kept a daily diary for 1-week, in which they recorded and described (for verification) each of their flashbacks, i.e., spontaneously occurring image-based intrusions of the trauma film (based on [24]). On return to the laboratory 1-week later, participants completed a recognition memory task to index voluntary memory retrieval. The task comprised a series of 32 written statements regarding the film, presented individually (e.g. ‘Three cars were involved in the crash’). Participants responded to each statement with either “true” or “false” and scored one point for each correct response. Finally, participants completed the Impact of Events Scale [40]–a clinical measure of PTSD symptomatic response adapted to the trauma film over the past 1-week.

## References

[1] American Psychiatric Association (2000). Diagnostic and Statistical Manual of Mental Disorders.

[2] Brewin CR, Holmes EA (2003). Psychological theories of posttraumatic stress disorder.. Clinical Psychology Review.

[3] Bryant RA, Harvey AG (2000). Avoidant coping style and post-traumatic stress following motor vehicle accidents.. Behaviour Research and Therapy.

[4] Pitman RK, Sanders KM, Zusman RM, Healy AR, Cheema F (2002). Pilot Study of Secondary Prevention of Posttraumatic Stress Disorder with Propranolol.. Biological Psychiatry.

[5] Henry R, Fishman JR, Youngner SJ (2007). Propranolol and the prevention of post-traumatic stress disorder: Is it wrong to erase the “sting” of bad memories?. The American Journal of Bioethics.

[6] Anderson MC, Green C (2001). Suppressing unwanted memories by executive control.. Nature.

[7] Hertel PT, Calcaterra G (2005). Intentional forgetting benefits from thought substitution.. Psychonomic Bulletin & Review.

[8] Depue BE, Curran T, Banich MT (2007). Prefrontal regions orchestrate suppression of emotional memories via a two-phase process.. Science.

[9] Holmes EA, Moulds ML, Kavanagh D (2007). Memory suppression in PTSD treatment [Letter to the Editor].. Science.

[10] National Institute for Health and Clinical Excellence (2005). Post-traumatic stress disorder (PTSD): the management of PTSD in adults and children in primary and secondary care..

[11] McNally RJ, Bryant RA, Ehlers A (2003). Does early psychological intervention promote recovery from posttraumatic stress?. Psychological Science in the Public Interest.

[12] Mayou RA, Ehlers A, Hobbs M (2000). Psychological debriefing for road traffic accident victims: Three-year follow-up of a randomized controlled trial.. British Journal of Psychiatry.

[13] Baddeley AD (2003). Working memory: looking back and looking forward.. Nature Reviews Neuroscience.

[14] Nader K (2003). Memory traces unbound.. Trends in Neurosciences.

[15] Walker MP, Brakefield T, Hobson JA, Stickgold R (2003). Dissociable stages of human memory consolidation and reconsolidation.. Nature.

[16] Ehlers A, Hackmann A, Michael T (2004). Intrusive re-experiencing in post-traumatic stress disorder: Phenomenology, theory, and therapy.. Memory.

[17] Holmes EA, Grey N, Young KAD (2005). Intrusive images and “hotspots” of trauma memories in posttraumatic stress disorder: An exploratory investigation of emotions and cognitive themes.. Journal of Behavior Therapy and Experimental Psychiatry.

[18] Andrade J, Kavanagh DJ, Baddeley A (1997). Eye-movements and visual imagery: A working memory approach to the treatment of post-traumatic stress disorder.. British Journal of Clinical Psychology.

[19] Baddeley AD, Andrade J (2000). Working memory and the vividness of imagery.. Journal of Experimental Psychology-General.

[20] Kavanagh DJ, Freese S, Andrade J, May J (2001). Effects of visuospatial tasks on desensitization to emotive memories.. British Journal of Clinical Psychology.

[21] van den Hout M, Muris P, Salemink E, Kindt M (2001). Autobiographical memories become less vivid and emotional after eye movements.. British Journal of Clinical Psychology.

[22] Horowitz MJ (1969). Psychic trauma. Return of images after a stressful film.. Archives of General Psychiatry.

[23] Holmes EA, Bourne C (2008). Inducing and modulating intrusive emotional memories: A review of the trauma film paradigm.. Acta Psychologica.

[24] Holmes EA, Brewin CR, Hennessy RG (2004). Trauma films, information processing, and intrusive memory development.. Journal of Experimental Psychology: General.

[25] Stuart ADP, Holmes EA, Brewin CR (2006). The influence of a visuospatial grounding task on intrusive images of a traumatic film.. Behaviour Research and Therapy.

[26] Weschsler D (1981). WAIS-R manual.

[27] Green SC, Bavelier D (2003). Action video game modifies visual selective attention [letter to the editor].. Nature.

[28] Haier RJ, Siegel BV, MacLachlan A, Soderling E, Lottenberg S (1992). Regional glucose metabolic changes after learning a complex visuospatial/motor task: a positron emission tomographic study.. Brain Research.

[29] Sims VK, Mayer RE (2002). Domain specificity of spatial expertise: The case of players.. Applied Cognitive Psychology.

[30] Stickgold R, Malia A, Maguire D, Roddenbury D, O'Connor M (2000). Replaying the game: Hypnagogic images in normals and amnesics.. Science.

[31] Hartshorne JK (2008). Visual working memory capacity and proactive interference.. PLoS ONE.

[32] Anderson CA, Gentile DA, Buckley KE (2007). Violent Video Game Effects on Children and Adolescents: Theory, Research and Public Policy.

[33] Wilper AP, Woolhandler S, Lasser KE, McCormick D, Cutrona SL (2008). Waits to see an emergency department physician: U.S. trends and predictors, 1997–2004.. Health Affairs.

[34] Schafe GE, LeDoux JE (2000). Memory consolidation of auditory pavlovian fear conditioning requires protein synthesis and protein kinase A in the amygdala.. The Journal of Neuroscience.

[35] Rudy JW (2008). Destroying memories to strengthen them.. Nature Neuroscience.

[36] Lee JLC (2008). Memory reconsolidation mediates the strengthening of memories by additional learning.. Nature Neuroscience.

[37] Dudai Y (2006). Reconsolidation: the advantage of being refocused.. Current Opinion in Neurobiology.

[38] Brunet A, Orr SP, Tremblay J, Robertson K, Nader K (2008). Effect of post-retrieval propranolol on psychophysiologic responding during subsequent script-driven traumatic imagery in post-traumatic stress disorder.. Journal of Psychiatric Research.

[39] The PC version of Tetris (copyright 1985–2008, Tetris Holdings LLC.) was obtained from Blue Planet Software (Honolulu, HI)

[40] Horowitz M, Wilner N, Alvarez W (1979). Impact of event scale: A measure of subjective stress.. Psychosomatic Medicine.

[41] Kavanagh DJ, Andrade J, May J (2005). Imaginary relish and exquisite torture: The elaborated intrusion theory of desire.. Psychological Review.

[42] Kemps E, Tiggerman M, Woods D, Soekov B (2004). Reduction of food cravings through concurrent visuospatial processing.. International Journal of Eating Disorders.

[43] Conway MA, Pleydell-Pearce CW (2000). The construction of autobiographical memories in the self- memory system.. Psychological Review.

[44] Dalgleish T (2004). Cognitive approaches to posttraumatic stress disorder: the evolution of multirepresentational theorizing.. Psychological Bulletin.

[45] Ehlers A, Clark DM (2000). A cognitive model of posttraumatic stress disorder.. Behaviour Research and Therapy.

[46] Gunter RW, Bodner GE (2008). How eye movements affect unpleasant memories: Support for a working memory account.. Behaviour Research and Therapy.

[47] Lilley SA, Andrade J, Turpin G, Sabin-Farrell R, Holmes EA (in press). Visuo-spatial working memory interferes with recollections of trauma.. British Journal of Clinical Psychology.

[48] Kemps E, Tiggemann M (2007). Modality-specific imagery reduces cravings for food: an application of the elaborated intrusion theory of desire to food craving.. Journal of Experimental Psychology: Applied.

